# Toxicity of aqueous mixture of phenol and chlorophenols upon photosensitized oxidation initiated by sunlight or vis-lamp

**DOI:** 10.1007/s11356-018-1286-x

**Published:** 2018-01-26

**Authors:** Magdalena Foszpańczyk, Emilia Drozdek, Marta Gmurek, Stanisław Ledakowicz

**Affiliations:** Faculty of Process and Environmental Engineering, Lodz University of Technology, Wolczanska 213, 90-924 Lodz, Poland

**Keywords:** Sunlight, Photodegradation, Immobilization, Chlorophenols, Toxicity

## Abstract

It is well established that aquatic wildlife in marine and freshwater of the European Union is exposed to natural and synthetic endocrine disruptor compounds (EDCs) which are able to interfere with the hormonal system causing adverse effects on the intact physiology of organisms. The traditional wastewater treatment processes are inefficient on the removal of EDCs in low concentration. Moreover, not only the efficiency of treatment must be considered but also toxicological aspects. Taking into account all these aspects, the main goal of the study was to investigate the photochemical decomposition of hazardous phenolic compounds under simulated as well as natural sunlight from the toxicity point of view. The studies were focused on photodegradation of 2,4-dichlorophenol as well as mixture of phenol, 2-chlorophenol and 2,4-dichlorophenol. Photosensitized oxidation process was carried out in homogeneous and heterogeneous system. *V. fischeri* luminescence inhibition was used to determine the changes of toxicity in mixture during simulated and natural irradiation. The photodegradation was carried out in three kinds of water matrix; moreover, the influence of presence of inorganic matter on the treatment process was investigated. The experiments with natural sunlight proved applicability of photosensitive chitosan for visible-light water pollutant degradation. The results of toxicity investigation show that using photosensitive chitosan for visible-light, the toxicity of reaction mixture towards *V. fischeri* has significantly decreased. The EC_50_ was found to increase over the irradiation time; this increase was not proportional to the transformation of the parent compounds.

## Introduction

Chlorophenols (CPs) are a group of compounds that have a dangerous effect on the environment, especially aqueous one. CPs are common contaminants which could be found in surface, ground, and drinking water (Davì and Gnudi 1999; Czaplicka 2004; Olaniran and Igbinosa 2011; Anku et al. 2017). Moreover, CPs were detected in wastewater as well as in sludge (Olaniran and Igbinosa 2011; Xing et al. 2012; Anku et al. 2017). Widespread occurrence of CPs is mostly associated with their application as pesticides in agriculture and industry; nevertheless, their usage as wood preservatives, personal care, and other products has also influence on environment (Davì and Gnudi 1999; Czaplicka 2004; Olaniran and Igbinosa 2011; Anku et al. 2017). The high toxicity especially to aqueous organisms’ ability to bioaccumulation and resistance to degradation makes the presence of CPs in the environment disturbing (Zona et al. 1999; Czaplicka 2004; Olaniran and Igbinosa 2011; Xing et al. 2012; Anku et al. 2017). Furthermore, during drinking water chlorine disinfection, the CPs may be formed when phenolic compounds are present in treated water (Michałowicz 2005; Michałowicz and Duda 2007; Chen et al. 2016; Anku et al. 2017). It should be noticed that CPs in very low concentration (even in ppb) are very odorous, taints the water, and changes its taste.

According to literature, CPs represent a public health concern due to their estrogenic, mutagenic, or/and carcinogenic effects (Michałowicz and Duda 2007; Xing et al. 2012; Igbinosa et al. 2013; Kadmi et al. 2015; Ge et al. 2017; Anku et al. 2017). The World Health Organization (WHO) has defined the maximum admissible concentration (MAC) in drinking water of 10 μg/L for 2-chlorophenol (2CP) and 40 μg/L for 2,4-dichlorophenol (2,4DCP) (Davì and Gnudi 1999; WHO 2011). The Environmental Protection Agency (US-EPA) includes phenol (PhOH), 2CP, and 2,4DCP in the priority pollutant list in relation with their toxicity degree, while 2CP is also listed on European Union (EU) dangerous list based on its toxicity, stability, and bioaccumulation (Igbinosa et al. 2013).The most often detected CPs in water are 2CP and 2,4DCP. Their concentration is very variable and depends on the type of water (e.g., in wastewaters, the detected values ranged from ng/L to even to mg/L (Czaplicka 2004; Limam et al. 2010; Olaniran and Igbinosa 2011)).

This article presents the study of photodegradation of the listed compounds and mixtures of phenol, 2-chlorophenol, and 2,4-dichlorophenol carried out in the heterogeneous photosensitized oxidation. The decomposition of the compounds with the usage of natural and simulated sunlight, as well as various types of water (demineralized, natural, and tap water) has been compared. Moreover, the toxicity of compounds and the effect of ternary mixture photodegradation on the toxicity has been examined which allowed to specify whether the method of photosensitized oxidation is able to significantly reduce the toxicity of the compounds treated.

## Material and methods

### Materials

All chemicals were of analytical grade. Phenol (PhOH) (≥ 99%) was purchased from CHEMPUR, Poland. 2-chlorophenol (2CP) was obtained from POCH, Poland. 2,4-dichlorofenol (2,4DCP) (≥ 99%) and dye sensitizers Al (III) phthalocyanine chloride tetrasulfonic acid (AlPcS_4_) (PC) were obtained from Frontier Scientific while chitosan from crab (degree deacetylation ≥ 75%) was purchased from Sigma-Aldrich, Germany. Na_2_CO_3_ (≥ 99%) and NaHCO_3_ (p.a.) were purchased from Sigma-Aldrich (Germany) and POCH (Poland) respectively.

### Methods

#### Photodegradation experiments

The study was conducted in a semi-continuous system in glass reactor of the volume 0.6 L (working volume 0.5 L) equipped with a porous plate to disperse air into the reaction solution. All the photochemical experiments were aerated and mixed by air bubbling. Two kinds of light source were used. As a light source, natural and simulated sunlight (high pressure sodium (HPS) grow lamp from Lumatek) was applied. During experiments with lamp, the temperature mixture was controlled using cooling jacket maintaining 20 °C, while with natural sunlight, the temperature was not forced.

The heterogeneous photosensitized oxidation was employed as a photochemical process. The photoactive chitosan containing AlPcS_4_ was used as an insoluble carrier. The chitosan solution carrier was prepared according to the procedure published elsewhere (Gmurek et al. 2017). When photoactive chitosan carrier was put into the reactor, the reaction solution was added.

Sunlight irradiation experiments were performed in sunny days (June/July 2016 and repeated on June/July 2017 humidity 75–85%, temperature 31–33 °C). The spectrum of light was collected with an Ocean Optics USB4000 fiber optic spectrometer with an approximate resolution of 0.4 nm (Ocean Optics Inc., USA).

Each experiment has been repeated at least twice. The presented results are average values obtained from both series of experiments (error was from 2 to 6%).

#### Water matrix characterization

The singly photodegradation was performed in demineralized water from the Millipore Milli-Q Plus System (MQ, 18.2MΩ) (pH 6.3–6.5). Ternary mixture (PhOH, 2CP, 2,4DCP) was additionally prepared in tap water (about pH 5) and natural water (pH 5.5) from a pond in Pabianice (Poland, 51° 38′ 50.3″ N 19° 22′ 19.7″ E). None of the pre-treatment process for MQ, tap, and natural water before photodegradation was done.

#### Analytical methods

The reaction progress was monitored by determination of phenol and chlorophenol concentration using an Agilent 1220 Infinity LC HPLC apparatus equipped with Poroshell 120 C18 column (2.7 μm) (Agilent Technologies Inc., Germany). The methanol with 0.1% formic acid (A) and water with 0.1% formic acid (B) were used as eluents. The method involved gradient elution with flow rate 0.8 ml/min. The gradient followed by from 0.00 to 1.00 min 5% A, than fast changed in 1.01 min to 60% A and kept till 10 min. The injection volumes for all samples were 70 μl, and all the compounds were monitored with a DAD detector. The detection wavelength was 280 nm for phenol (PhOH) and 2,4-dichlorophenol (2,4DCP). For 2-chlorophenol, the monitoring wavelength was 273 nm. The limit of detection and limit of quantification were determined by signal-to-noise method and these were observed above 0.03 and 0.07%, respectively, for all compounds. Under these conditions, excellent linearity was obtained (r^2^ = 0.999) for each compounds. The compounds in ternary mixture were analyzed in one injection, in which they were characterized by its respective retention time. The peaks were well separated under the above analysis conditions mentioned above. Before injection, the samples were filtered by 0.45 μm NY filter.

The anion concentrations in water matrixes were determined by an ion chromatograph (ICS-1100, DIONEX, USA) on an IonPac AS23 (with temperature 35 °C). The 0.45 mM Na_2_CO_3_ and 0.8 mM NaHCO_3_ were used as eluents with the flow rate 0.3 ml/min. The injection volume was equal 5 μl.

#### Ecotoxicity bioassay

The acute ecotoxicity bioassay was conducted using a Microtox Model 500 analyzer (Modern Water, New Castle, DE, USA) with the marine bacterium *Vibrio fischeri* as a bioluminescent indicator. The “81.9% basic test” protocols available with the MicrotoxOmni™ analyzer software were used for the ecotoxicity assessment of samples. The toxicity test has been expressed as effective concentration (EC_50_), its mean toxicant causing 50% inhibition of the luminescence as well as Toxic Unit according to calculation presented in (Persoone et al. 2003).

## Results and discussion

In order to demonstrate the effect of photochemical processes on aqueous solution toxicity containing phenolic compounds such as phenol, 2-chlorophenol, and 2,4-dichlorophenol at the beginning, the investigation was focused on photodegradation of the individual compounds under simulated visible radiation. Direct photolysis and heterogeneous photosensitized oxidation with photoactive chitosan carrier with an immobilized photosensitizer (AlPcS_4_) in the form of a spherical hydrogel have been carried out.

**Fig. 1:**
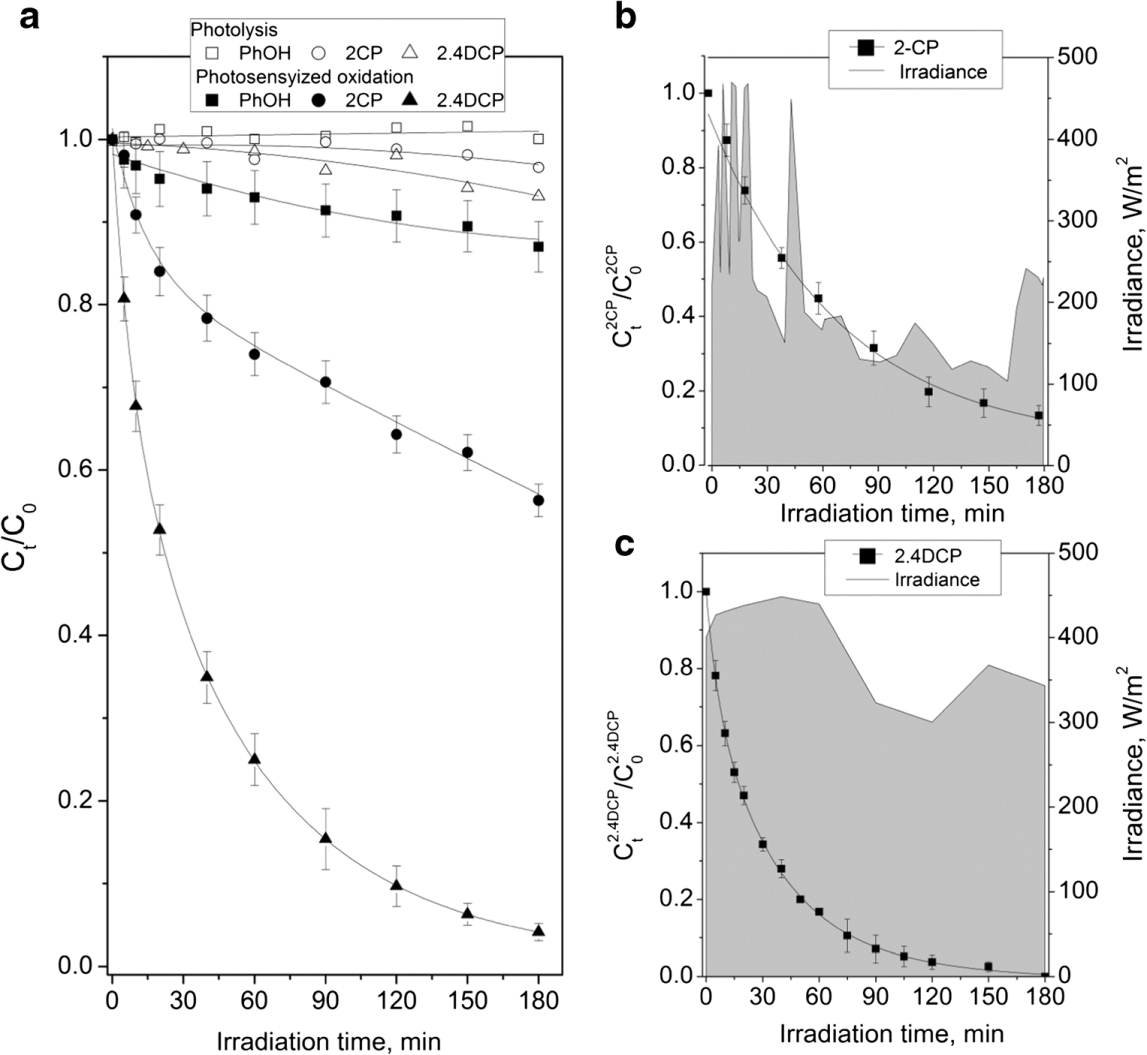
Photodegradation of phenol and chlorophenols (singly) under simulated (**a**) and natural sunlight (**b**). **c** In the presence of photoactive chitosan beads (C_0_ = 10 mg/L, m_CH_ = 55 g, lamp 250 W, MQ)

As can be seen in Fig. 1a, a negligible photolysis of PhOH, 2CP as well as 2,4DCP is observed during 3 h of irradiation. While at the same time, the 10, 46, and almost 100% of PhOH, 2CP, and 2,4DCP concentration was removed during photosensitized oxidation, respectively. The experiments with sodium azide, well-known singlet oxygen (^1^O_2_) quencher, proved that ^1^O_2_ is responsible for phenol degradation (data not shown). This was in agreement with previous study in homogenous and heterogeneous photosensitized oxidation of 2,4DCP (Gryglik et al. 2016; Gmurek et al. 2017). It must be mentioned that phenolic compounds undergo faster degradation via ^1^O_2_ in dissociated form (Gryglik et al. 2004; Miller 2005; Gmurek et al. 2017). All experiments were performed in Milli Q water (pH ~ 6.3–6.5) wherein PhOH exists in undissociated form. Furthermore, the fraction of dissociated molecules of chlorophenols increases with increasing chlorine substituent. Moreover, the single chlorophenol degradation has been investigated under natural sunlight. The intensity of sunlight was collected during all experiments and the changes of it are presented in Fig.1b, c. It is evidence that photodegradation of both (2CP and 2,4DCP) chlorophenols occurs much faster under natural sunlight (Fig.1b, c).

Based on performed experiments, toxicity analysis has been done (Table.1). The toxicity of reaction solutions was determined by using Microtox® toxicity test, which based on using the marine luminescent bacterium *Vibrio fischeri*. This bacterium emits light as a result to metabolic process catalyzed by enzyme luciferase (Miyashiro and Ruby 2012; Baran and Tarnawski 2013). Exposure to the contaminants may disrupt these processes and lead to reduction of luminescence, which is taken as a measure of toxicity. Based on the toxicity test performed before the photodegradation, the EC_50_ value for PhOH, 2CP, and 2,4DCP was determined (Table.1).

**Table 1:** Toxicity assessment during photodegradation

	EC_50_ (mg/L)		TU^c^				
	This study	references	0 min	20 min	60 min	120 min	180 min
PhOH	20.22	21-24^a^	1.04 (Cl. III)	–	–	–	1.9 (Cl. III)
2CP	15.15	16.1–69.5^a,b^	1.16 (Cl. III)	–	6.57 (Cl. III)	12.41 (Cl. IV)	9.28 (Cl. III)
2,4DCP	2.93	1.24–6.06^a,b^	10.52 (Cl. IV)	12.97 (Cl. IV)	19.42 (Cl. IV)	15.27 (Cl. IV)	8.4 (Cl. III)

^a^Kaiser and Palabrica 1991

^b^Zona et al. 1999

^c^Persoone et al. 2003

The highest toxicity towards *V. fischeri* exhibits 2,4DCP. It is five and seven times more toxic than 2CP and PhOH, respectively. It should be noticed that the EC_50_ determined in this study is consistent with literature values (Kaiser and Palabrica 1991; Zona et al. 1999). As it is presented in Table 1, all reaction solution before treatment reveals acute toxicity (PhOH, 2CP) or high acute toxicity (2,4DCP).

Alternative form of expression of toxicity is the Toxic Unit (TU, (Persoone et al. 2003)). The toxicity of PhOH solution after the photodegradation process has increased. After 3-h treatment, the TU of the PhOH reaction solution increased from 1.04 to 1.94 (almost twice), despite of only 10% of PhOH reduction. It is probably due to the formation of pyrocatechol, hydroquinone, or benzoquinone, which are much higher toxic compounds than PhOH (Santos et al. 2004; Grabowska et al. 2012).

Furthermore, although the photodegradation of 2CP proceeds faster than PhOH (~ 50% reduction after 3 h), the higher toxicity after treatment was observed. The TU for 2CP increased almost nine times (from 1.06 to 9.28) after 3 h of photodegradation. It is well known that pyrocatechol (EC_50_^*V.fischeri*^ = 8.32 mg/L (Santos et al. 2004)) is a product of the photodegradation of undissociated 2CP (Czaplicka 2006). Moreover, the study allowed us to confirm the identification of 2-chloro-1,4-benzoquinone (EC_50_^*V.fischeri*^ = 0.03 mg/L (Ortiz de García et al. 2014)), 2-chlorohydroquinone, and maleic acid (EC_50_^*V.fischeri*^ = 247 mg/L (Santos et al. 2004) as phototransformation products of 2CP using ^1^O_2_.

Surprisingly, only during 2,4DCP photodegradation the toxicity decreases. After 3 h of treatment, almost all 2,4DCP was removed and the TU decreased from 10.52 to 8.4. However, it should be noticed that during treatment, the toxicity that was firstly increasing, finally decreased, probably due to the fact that during photodegradation, more toxic transformation products were formed and then degraded. Monochlorophenol, 4-chlorocyclopentadienyl carboxylic acid, chlorohydroquinone, and 4-chloro-1,2-benzenediol were main well-known 2,4DCP phototransformation products (Czaplicka 2006). During this study, 2CP was detected. Furthermore, release of chloride to the aqueous solution was observed.

The study confirmed that application of visible light for decontamination of highly chlorinated phenols is more rapid than the degradation of the less-chlorinated phenols (Chung et al. 2013), or phenol, that was approved by the presence of residual chlorinated compounds and released chloride ions.

**Fig. 2:**
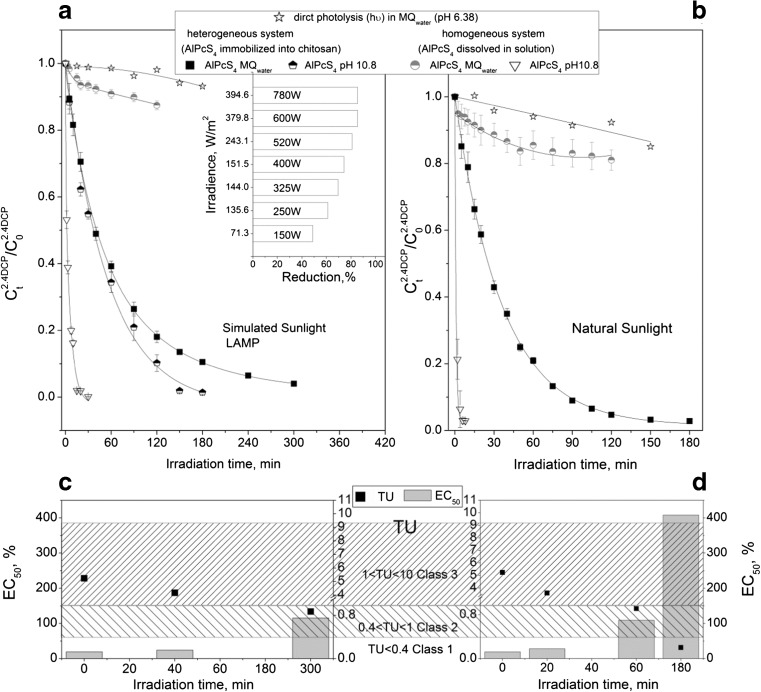
Photodegradation of 2,4DCP under simulated and natural sunlight in the presence of photoactive chitosan beads and the toxicity assessment (C_2,4DCP_ = 19 mg/L, m_CH_ = 55 g, *T* = 20 °C, lamp 250 W). Insert: Influence of 60 min. Irradiance on photodegradation of 2,4DCP

Based on the above experiments, the next step was to investigate the comparison of the influence of simulated and natural sunlight on the most toxic 2,4DCP. Figure 2a, b shows the decay of 2,4DCP in MQ solution under natural and simulated sunlight in the presence of photoactive chitosan (to achieve the most comparable data, the same reaction solution was used for both processes). Moreover, to compare the process in heterogeneous (photosensitizer immobilized into chitosan beans) as well as in homogeneous (photosensitizer dissolved in reaction solution) system, photosensitized oxidation of 2,4DCP was performed.

As can be seen, the photosensitized oxidation in homogeneous system occurred very fast in alkaline solution (pH 10.8, dissociated form of contaminant but unreal condition to find in the environment), while in MQ water (pH 6.3–6.5), where phenol and chlorophenols are in undissociated form, the reaction is unhurried. While in heterogeneous system, the reaction in MQ water was comparable with the reaction in pH 10.8 (Fig.2a). Presumably, increasing reaction rate in heterogeneous system is connected with simultaneous photoreaction and adsorption on chitosan carrier.

As can be seen, both photolysis and photosensitized oxidation occur faster in case of reaction solutions irradiated by natural sunlight (Fig. 2a, b) that is mainly due to the presence of UV radiation in the natural sunlight spectrum. Application of photoactive chitosan accelerated 2,4DCP degradation. However, in order to achieve almost completely 2,4DCP removal (96% degradation), the solution was 5 h irradiated by simulated sunlight, while 3 h was enough to achieve 98% degradation under natural sunlight (Fig. 2a, b). As demonstrated in Fig. 2a insert, the same photodegradation efficiency can be reached when irradiance was higher than 151 W/m^2^.

As has been pointed out in Fig.2c, d, the used of light source affects the toxicity. Based on the TU at the beginning, the reaction solution exhibited acute toxicity (Class III). The comparable results were attainted after 40 min of simulated sunlight irradiation and 20 min of natural sunlight irradiation. After 60 min and 300 min under natural sunlight and simulated sunlight irradiation, respectively, the reaction mixture showed slight acute toxicity (Class II). Furthermore, after 180 min of photosensitized oxidation with application of photoactive chitosan, reaction solution demonstrated no acute toxicity (Class I). It can be concluded that when natural sunlight is applied not only the costs of the treatment is lower but also the toxicity of the reaction solution decreases much faster, and the possibility of less toxic transformation products can be formed.

**Fig. 3:**
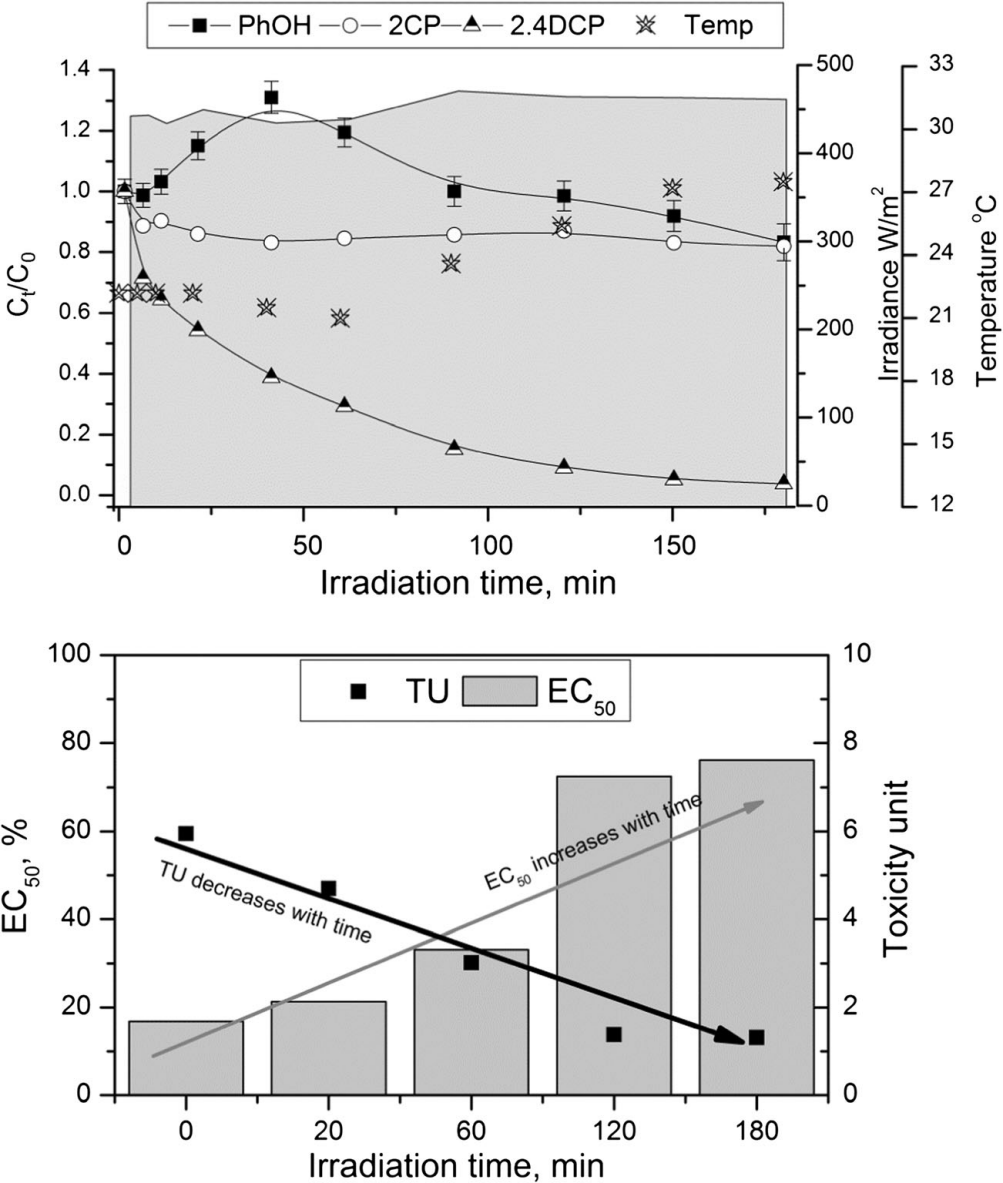
Decay (**a**) and toxicity assessment (**b**) of photodegradation of ternary mixture (PhOH, 2CP, and 2,4DCP) under natural sunlight in the presence of AlPCS_4_ immobilized into chitosan beads (C_PhOH_ = 8.6 mg/L; C_2CP_ = 5.4 mg/L; C_2,4DCP_ = 16 mg/L, MQ, m_CH_ = 55 g)

**Fig. 4:**
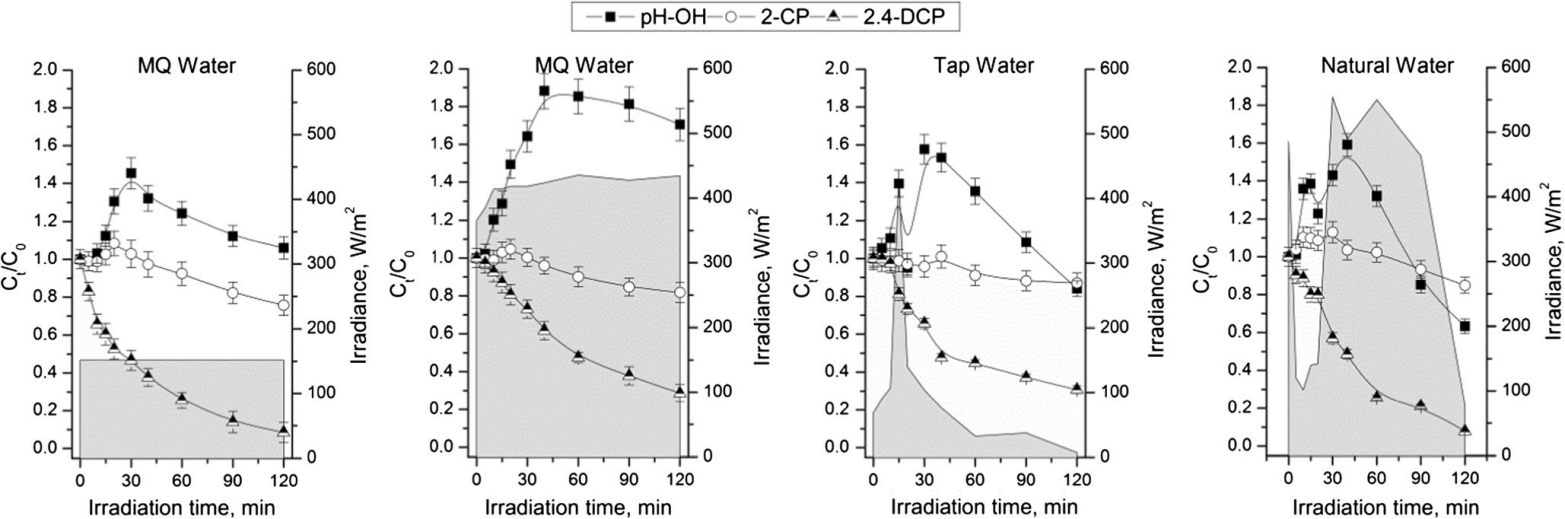
Photosensitized oxidation of ternary mixture (PhOH, 2CP, and 2,4DCP) under simulated (the left side merge) and natural sunlight (C_0_ = 10 mg/L, m_CH_ = 55 g, Lamp 400 W) in different water matrix

In the next step of investigation, the heterogeneous photosensitized oxidation of ternary mixture (PhOH, 2CP, and 2,4DCP) was examined. Based on singly photodegradation, two kinds of experiments were carried out. Firstly, the domination of 2,4DCP was checked in the mixture (mass concentration proportion 2,4DCP/2CP/PhOh was equal 1:0.4:0.6, Fig.3). The 2CP concentration was the lowest due to the most harmful toxicity results from singly photodegradation. Secondly, the equal concentration of each contaminant was applied (Fig.4).

As can be seen in Fig. 3, after 3 h of sunlight irradiation, almost completely (96%) degradation of 2,4DCP was observed, despite its high initial concentration. While 2CP as well as PhOH concentrations were continually changing during treatment, finally, after 3 h, only 20% removal of 2CP was obtained. It is worth to notice an increase of PhOH concentration, which indicates that it is a transformation product of chlorophenol decomposition. The same finding was made in case of 2CP, which is the transformation product of 2,4DCP degradation. It was also interesting that the slope of 2,4DCP concentration decay (which is the measure of degradation rate) was almost the same in the component mixture as well as in singly photodegradation. It should be also noticed that temperature only slightly increased during photodegradation. However, based on previous research in the case of 2,4DCP, the temperature has not so much influence on photosensitized oxidation (Olak-Kucharczyk et al. 2017).

In the case of photosensitization of the ternary mixture (PhOH, 2CP, and 2,4DCP), it is clear that the EC_50_ values shown in Fig.3 have consistently increased. Taking into account of individual concentrations in solution (nearly all of the 2,4DCP has been degraded), the most toxic compound was removed, while 2CP which was the most harmful singly degraded has remained in solution and only slightly degraded. Surprisingly, there was no increase in toxicity at any stage of the process, which may indicate that not so toxic byproducts were produced as in singly degradation or that they were produced in amounts that did not affect the overall toxicity of the solution. Moreover, the TU decreased from 5.9 to 1.3, notwithstanding the solution still is classified as acute toxic. Nevertheless, it can be assumed that the use of sunlight contributes in some way to the formation of less toxic products.

Analyzing the results of the second approach (equal concentration of each contaminants), it can be seen that still the fastest degradation was achieved for 2,4DCP. The differences in 2,4DCP degradation efficiency between simulated and natural sunlight irradiation were observed (Fig.4); much more visible is the trend in PhOH concentration change during photodegradation. After sunlight photodegradation, still, 80% of 2CP remained in the solution, but PhOH concentration was higher than in the case of lamplight. However, there was no significant influence on the toxicity of the postreaction mixtures.

The influence of the water matrix on the ternary mixture natural sunlight photodegradation has been checked with water matrix MQ, tap, and natural water. Ion characterization of water matrixes is presented in Table 2. As can be seen in Fig. 4, the photodegradation of the mixture depends on water matrix; however, the same trends were observed (2,4DCP is the fastest degraded, and the increased concentrations of 2CP as well as PhOH increased). It can be assumed, that the pathway of the photodegradation is a little bit different. Almost all 2,4DCP was removed when natural water was used, while similar efficiency was observed when MQ and tap water was applied. On the other hand, the improvement of 2CP degradation was not observed when natural or tap water was used. When PhOH photodegradation in tap or natural water is considered, the meaningful changes were observed in comparison to MQ water photodegradation.

**Table 2:** Ion characterization of water matrix

Ion	F^−^	Cl^−^	Br^−^	NO_2_^−^	NO_3_^−^	PO_4_^3−^	SO_4_^2−^
Unit	(mg/L)						
MQ Water	0.017	0.079	0.075	0.059	0.121	0.146	0.075
Tap Water	0.300	10.961	0.082	0.047	3.357	0.200	27.536
Natural Water	0.293	14.331	0.085	0.061	0.109	0.247	38.562

As can be seen in Table 2, the differences in the ion characterization between three water matrices are observed in the concentration of chlorine, nitrate, sulfate ions, and only slightly for phosphate ion. It is well known that nitrate and chloride ions as well as humic acids have strong influence on photodegradation (Ge et al. 2009; Chen et al. 2013). The photodegradation in the presence of those ions can change the mechanism of decontamination. It should be noticed that experiments with sodium azide and *t-*BuOH excluded hydroxyl radical pathway. Moreover, the experiments of 2,4DCP photodegradation with inherence of chlorine, nitrate, and phosphate ions did not influence on photodegradation process (data not shown). Based on this finding, it could be concluded that for acceleration of photodegradation in natural water, dissolved organic matter is responsible. The role of humic acids in photodegradation of aqueous contaminant was studied by Chen et al. (2013) and Gmurek et al. (2015) and confirmed that humic acids may play the significant role in intensification of photodegradation.

## Conclusions

The experiments under sunlight proved applicability of photosensitive chitosan for aqueous solution of single as well as mixture of phenol, 2-chlorophenol, and 2,4-dichlorophenol degradation. The contribution of photolysis in photosensitized oxidation (mostly via ^1^O_2_ mechanism) of the pollutants was negligible. The fastest degradation was achieved for 2,4DCP. The results of toxicity investigation show that toxicity of reaction mixture towards *V. fischeri* has significantly decreased in photooxidation progress. The EC_50_ was found to increase over the irradiation time; however, this increase was not directly related to the transformation of the parent compounds. The photodegradation of the phenol compound mixture depends on water matrix. In all water matrixes, 2,4DCP is the fastest degraded, while the increased concentrations of 2CP as well as PhOH were detected. The higher 2,4DCP photodegradation efficiency in the natural water was probably due to the dissolved organic as well as inorganic matter.
